# Addendum to Systematic Review of Remdesivir for the Treatment of COVID-19

**DOI:** 10.5811/westjem.2020.5.48121

**Published:** 2020-05-22

**Authors:** Arif Musa, Elizabeth Warbasse, David A. Baron, Kasim Pendi, Areio Hashemi, Jenna Yousif, Emily Blodget, Susan Stevens, Besma Aly, Alisha Khambati, Sarkis Kouyoumjian

**Affiliations:** * Wayne State University School of Medicine, Detroit, Michigan; † Musa Biomedical Consulting, LLC, Anaheim, California; ‡ Western University of Health Sciences, Office of the Provost, Pomona, California; § Southern California University of Health Sciences, School of Professional Studies, Whittier, California; ¶ William Carey University, College of Osteopathic Medicine, Hattiesburg, Mississippi; || University of Southern California Keck School of Medicine, Division of Infectious Diseases, Department of Medicine, Los Angeles, California; # Wayne State University School of Medicine, Department of Emergency Medicine, Detroit, Michigan

To the Editor:

We are honored to have our systematic review of remdesivir for the treatment of COVID-19 published in the *Western Journal of Emergency Medicine*.^1^ Recently, new studies regarding the use of remdesivir have prompted us to submit this letter. On April 10, Grein et al reported that most patients given remdesivir in an open-label program exhibited observable clinical improvement.^2^ However, Wang et al did not find a statistically significant benefit with remdesivir in a randomized, double-blind, placebo-controlled trial of 237 patients that was published on April 29.^3^ On the same day, a press release by the National Institute of Allergy and Infectious Diseases (NIAID) regarding the Adaptive COVID-19 Treatment Trial (ACTT) reported significantly reduced time to recovery and mortality with remdesivir.^4^ Therefore, the purpose of our letter is to briefly analyze these new findings and determine whether additional conclusions in remdesivir’s treatment of COVID-19 can be drawn.

Grein et al analyzed open-label data from 61 patients who were treated with remdesivir between January–March 2020. Patients were administered a loading dose of 200 milligrams (mg) on the first day followed by nine days of 100 mg infusions. Clinical improvement was based on a six-point scale: 1 = not hospitalized; 2 = hospitalized but not requiring supplemental oxygen; 3 = hospitalized and requiring supplemental oxygen; 4 = hospitalized and requiring nasal high-flow oxygen therapy, noninvasive mechanical ventilation or both; 5 = hospitalized requiring mechanical ventilation, extracorporeal membrane oxygen, or both. Most patients (68%) exhibited clinical improvement and 84% were discharged or showed a decrease of two points or more at follow-up. In total, 13% died and 60% exhibited adverse events, most commonly hepatic and renal dysfunction. Although these findings are encouraging, the small sample size, lack of a comparison group, case-by-case variation in supportive care, and missing data make it difficult to draw robust conclusions.

The first randomized, controlled clinical trial of remdesivir for treatment of COVID-19 was published in *The Lancet* on April 29. This study analyzed treatment of 237 patients (158 given remdesivir and 79 given placebo). Although the remdesivir group showed a reduced time to clinical improvement (18 vs 23 days), this was statistically insignificant. Neither mortality (14% vs 13%) nor adverse events (66% vs 64%) were commonly associated with remdesivir. The most common complications included constipation, hypoalbuminemia, hypokalemia, and anemia. However, there are several limitations to these results. Firstly, patients in the remdesivir and placebo groups received interferon alfa (29% vs 38%), lopinavir-ritonavir (28% vs 29%), antibiotics (90% vs 94%), and corticosteroids (65% vs 68%) before and after enrollment. These additional drugs make it difficult to differentiate between the effects of remdesivir and other treatments. Moreover, the placebo group received a higher percentage of these drugs. Secondly, 36 patients discontinued treatment due to adverse events, reducing the sample size. Since the trial was terminated early on March 29, the statistical power was reduced from 80% to 58%. Though these findings do not support remdesivir to treat COVID-19, the methodological limitations, missing data, and early termination moderate the results.

The ACTT is an ongoing randomized, double-blinded controlled trial of remdesivir that began enrolling on February 21.^5^ After, the data and safety monitoring board performed a preliminary analysis of 1063 patients, the NIAID reported on April 29 that remdesivir statistically significantly reduced time to recovery compared to placebo (11 vs 15 days, p<0.001). There was also a modest increase in survival (8.0% vs 11.6%), which approached statistical significance (p = 0.059). Although preliminary findings released by NIAID are supportive, it remains possible that the final results may differ at the conclusion of the trial. Also, the press release did not reveal any data about adverse events, loss to follow-up, or other complications. Nevertheless, on May 1, based on the ACTT and Gilead open-label trial, the US Food and Drug Administration issued an Emergency Use Authorization (EUA) for use of remdesivir for COVID-19.^2^,^4^,^6^ Physicians may find it difficult to make informed decisions regarding treatment of patients with COVID-19. Given the recent EUA, we recommend making the decision to administer remdesivir based on the highest quality of evidence in the literature, which suggests decreased time to recovery and the possibility of increased survival.
